# Molecular approach to describing a seed-based food web: the post-dispersal granivore community of an invasive plant

**DOI:** 10.1002/ece3.580

**Published:** 2013-04-28

**Authors:** Jonathan G Lundgren, Pavel Saska, Alois Honěk

**Affiliations:** 1 USDA-ARS, North Central Agricultural Research LaboratoryBrookings, South Dakota, 57006; 2 Crop Research InstitutePraha, Czech Republic

**Keywords:** Granivore, gut analysis, seed predation, *Taraxacum officinale*, trophic linkages

## Abstract

Communities of post-dispersal granivores can shape the density and dispersion of exotic plants and invasive weeds, yet plant ecologists have a limited perception of the relative trophic linkages between a seed species and members of its granivore community. Dandelion seeds marked with Rabbit IgG were disseminated into replicated plots in the recipient habitat (South Dakota) and the native range (Czech Republic). Arthropods were collected in pitfall traps, and their guts were searched for the protein marker using enzyme-linked immunosorbent assay (ELISA). Seed dishes were placed in each plot, and dandelion seed removal rates were measured. The entire experiment was repeated five times over the dandelion flowering period. Gut analysis revealed that approximately 22% of specimens tested positive for the seed marker. A more diverse granivore community had trophic linkages to seeds than has been previously realized under field conditions. This community included taxa such as isopods, millipedes, weevils, rove beetles, and caterpillars, in addition to the traditionally recognized ants, crickets, and carabid beetles. Rarefaction and Chao analysis estimated approximately 16 and 27 species in the granivore communities of the Czech Republic and South Dakota, respectively. *Synthesis*: Generalist granivore communities are diverse and polyphagous, and are clearly important as a form of biotic resistance to invasive and weedy plants. These granivore communities can be managed to limit population growth of these pests.

## Introduction

Post-dispersal granivory (i.e., granivory that occurs after the seeds leave the parent plant) affects the density and dispersion of plants within a habitat (Lundgren 2009). Daily seed removal rates of plants can be quite extensive (O'Rourke et al. 2006; Menalled et al. 2007), but are typically < 10% in most systems (Lundgren 2009). This species-rich community is typically regarded as being dominated by rodents (Hulme 2002; Westerman et al. 2003a), carabid beetles (Lundgren 2009), crickets (O'Rourke et al. 2006; Lundgren and Harwood 2012), and ants (MacMahon et al. 2000; Nicolai and Boeken 2012), but the community is dynamic in space and time which makes it difficult to generalize as to constituency in a particular habitat. Furthermore, each taxon within the community has preferences for certain seed characteristics such as seed size, defensive properties, seed coat strength, and chemical and nutritional attributes (Pizo and Oliveira 2001; Honěk et al. 2003; Lundgren and Rosentrater 2007). These preferences change in response to various physiological and environmental circumstances (Lundgren 2009). These preferences ultimately drive which species are consumed within a habitat.

Research on granivory as a form of weed management has emphasized identifying the impact of granivores on a seed resource rather than on identifying which granivores have strong trophic linkages to a particular seed. Many studies examine the numerical associations of specific granivore taxa with seed removal rates (Davis and Liebman 2003; O'Rourke et al. 2006; Saska et al. 2008; Bohan et al. 2011). Some studies reinforce these numerical correlations by restricting arthropod access to sentinel seed patches based on granivore size or seasonal phenology (Menalled et al. 2000; Baraibar et al. 2012), or by confirming consumption rates using laboratory assays that demonstrate that specific granivores consume a particular seed (Honěk et al. 2003, 2005; Saska 2008bb). These techniques are helpful in establishing the intensity of seed consumption and in narrowing the number of potentially important members of the granivore community. However, these techniques disrupt the normal behaviors of granivores, and therefore the relative strengths of trophic interactions between specific members of the granivore community remain elusive. One approach frequently used to establish trophic connections of animals to specific foods has been gut or fecal analysis that searches for a food-associated, often molecular, marker; this is particularly well studied for insect predators (Sheppard and Harwood 2005; Weber and Lundgren 2009). Surprisingly, little attention has been given to using this technique to examine the granivore community associated with specific plant species. An exception is in examining the diets of birds (Legagneux et al. 2007) and seed caches of rodents (Li and Zhang 2007), where scat analysis, forced regurgitation, microscopic gut analysis, and marking the seeds with a fluorescent or radioactive dust can establish trophic links to seeds with limited disruption of the normal feeding behavior of the granivore. Still, molecular gut analysis (searching for nucleic acids or proteins; the predominant methods used in insect predation studies) remains relatively undeveloped with respect to granivory.

Dandelion (*Taraxacum officinale* agg.; Asteraceae) is a perennial plant that is native to Eurasia and has invaded much of the rest of the world over the past 100–150 years (Stewart-Wade et al. 2002). In North America, dandelions are cosmopolitan in most landscapes with spring moisture, including perennial agroecosystems and no-till cropland, where this species has become a readily recognizable pest (Johnson and Larson 2007). In addition to competing with natives for light and space, the plant can compete for pollinator services with native flowers and possibly hybridize with native congeners (Kandori et al. 2009; Matsumoto et al. 2010; but see Jones 2004). Research on dandelions in the Old World has advanced our understanding of seed demography and ecology in plants, and the constraints that limit granivory on a target plant species (Brock et al. 2005; Honek and Martinkova 2005; Honěk et al. 2005, 2011; Martinková and Honěk 2008; Martinková et al. 2009). The post-dispersal granivore community in North America is entirely undescribed.

One problem with our understanding of how enemies of invasive species respond to invasive and weedy plants is that only a small number of studies have explicitly examined the enemy community in both the native and recipient regions (Liu and Stiling 2006). This research aims to advance technology (protein marking of seeds and gut analysis of putative predators, sensu Hagler 2006) to definitively describe the relative trophic linkages of dandelion seeds to the granivore communities in its native range (Czech Republic) and a recipient biota (South Dakota).

## Materials and Methods

### Field sites

Research was conducted in both the recipient biota (South Dakota) and the donor biotia (Czech Republic) of dandelion. In South Dakota (SD), research was conducted in 2009 and 2010 on the Eastern South Dakota Soil and Water Research Farm (44.353011, −96.799648; latitude, longitude) near Brookings, South Dakota. In the Czech Republic, research was conducted at the Crop Research Institute (50.511377, 14.188966) near Prague only in 2009 (efforts to capture insects failed in 2010). These regions have similar average high and low daily air temperatures in May and June (May average high 18.7–21.0°C, average low 7.0–8.4°C; June average high 22.0–26.0°C, average low 11.2–12.0°C) and precipitation levels (a monthly average of 73–107 mm in May and June). At both locations, the habitats were perennial orchards with mowed perennial ground cover (one site in the Czech Republic remained unmowed) with edge habitats that varied from tree lines to open meadows. Each had high densities of dandelions. In the Czech Republic, dandelions were abundant at all sites, but were not quantified. In South Dakota, mean (±SEM) densities of plants were 172 ± 20 and 330 ± 33 dandelions per m^2^ in 2009 and 2010, respectively. There were three replicate sites (each 100 m^2^; 10 × 10 m) in the Czech Republic that were separated by 150–300 m. Five replicate sites of the same size were selected in South Dakota, and these were at least 30 m apart.

### Insect community assessment

In the center of each plot (5 × 5 m), two sets of barrier-linked pitfall traps were placed perpendicular to one another, each occurring centrally in one half of the plot. Barrier-linked pitfall traps amplify trap captures over individual pitfalls; in our study, these consisted of two dry pitfall traps (10 cm diameter opening), spaced 1.5 m apart that were connected using a metal sheet (150.0 × 14.5 cm long × tall) held vertically to direct foraging arthropods into the traps at either end (Lundgren et al. 2009). Traps were activated by placing a glass collection jar with a 1 × 1 cm piece of material impregnated with insecticide (Dichlorvos, 2, 2-Dichlorovinyl dimethyl phosphate 18.6%; Prozap; Loveland Industries Inc., Greeley, CO). In the Czech Republic, four single traps per site were added to increase the catch size. These traps consisted of two nested plastic cups (opening diameter 7 cm, volume 300 ml, covered with a metal roof to avoid flooding), and each contained insecticide.

### Seed marking and dissemination

For this work, we adapted protein marking techniques developed by Hagler et al. for studying insect predation and movement (Hagler 2011; Slosky et al. 2012). In South Dakota, mature dandelion seeds were collected from plants near the study sites using a vacuuming leafblower (Poulan PRO® leafblower, Electrolux Home Products, Augusta, GA). Fresh seeds were collected each year of study from the earliest flowers; viability of collected seeds was not evaluated in the study. Seeds were returned to the laboratory, dried, and the pappus and debris were removed from the seed. Seeds for the experiment in the Czech Republic were purchased from Herbiseed®, Twyford, U.K. Cleaned seeds were kept at 10°C until they could be marked. In South Dakota, individual batches of cleaned seeds were marked (approximately 41 g; seeds from our research site weighed approximately 469 μg each which roughly confirms other studies; Hale et al. (2010) using a filtration approach. Each cohort of seeds was soaked in 75 mL 1 × phosphate-buffered saline (PBS) and 410 μL of rabbit IgG (Product number I8140; Sigma-Aldrich, St. Louis, MO) for 2 min. The samples were then air-dried at 25°C for approximately 12 h. In the Czech Republic, a quantity of 42 g of seeds (per cohort) was soaked in 100 mL of the PBS and 420 μg of rabbit IgG overnight at 5°C. Soaked seeds were then lyophilized (PowerDry LL3000, Thermo, Shanghai, China) for 24 h, at which point the seeds were dry. Seeds were spread within a few hours of being dried at a rate of 3500 seeds per m^2^ (A. H. determined this as an average seed density in the Czech Republic) evenly throughout the central region (5 × 5 m) of each plot at 08:00 on the first morning of each observation period. Granivory of marked seeds was examined over five temporal periods that spanned the flowering period of dandelion in each study year. In 2009, the South Dakota observation periods were initiated on May 22, June 2, 10, 16, and 23 and in the Czech Republic on May 12, 19, 26, and June 2 and 9. In 2010, observations were initiated in South Dakota on May 26, June 2, 8, 15, and 22. Pitfall traps were activated at 08:00 on the first day of the trial; they were checked twice daily (at 10:00 and 22:00 in South Dakota, and at 08:00 and 20:00 in the Czech Republic) for 48 h in the South Dakota and for 24 h in the Czech Republic. All arthropods collected were identified to species level and were frozen at −20°C within 30 min of collection. All specimens from the Coleoptera, Orthoptera (Ensifera and Caelifera), Diplopoda, Isopoda: Oniscidea, and Hymenoptera: Formicoidea had their gut contents analyzed for the presence of the protein marker; spiders, phalangiids, and centipedes were not advanced for gut analysis.

General seed removal rates of unmarked dandelion seeds were monitored using sentinel seed patches (each with 25 seeds) in each plot. In South Dakota, intact dandelion seeds were affixed to the bases of each of 125 plastic Petri dishes (10 cm diam) using double-sided tape. Fine quartz sand was placed around the seed to prevent insects from adhering to the tape. On the first day of each sample period, five dishes were placed on the soil surface in a centralized × pattern in each plot, and residue from surrounding soil was placed lightly over the tops. Seed dishes were recollected after 7 days (exceeding the pitfall trapping period, but not overlapping observation periods). In the Czech Republic, arthropod activity was monitored using seed cards (sand paper strips 3 × 8 cm, seeds per card glued with 3M® repositionable glue; Westerman et al. 2003b). Five cards per site were held in place with nails and were exposed for 1 day following the deposition of marked seeds. The mean numbers of seeds removed or damaged per sentinel station were counted for each plot and observation period.

The ability to detect the protein marker on the seeds over time was evaluated by exposing small satchels of marked seeds to the environment. Seed satchels consisted of IgG-marked seeds (25 g) in a coarse muslin material folded in half. Five satchels were affixed to the soil in each plot with a small metal stake. Satchels were recovered 0, 2, 5, 7, and 9 days after placement, and were held at −20°C until processing. To remove the marker, three subsamples of five seeds from each satchel were placed into individual 1.5 mL microcentrifuge tubes containing 500 μL of 1 × PBS. These subsamples were incubated at 10°C for 24 h. Each of these subsamples was analyzed using the enzyme-linked immunosorbent assay (ELISA) procedures outlined below.

### ELISA protocols

Insects were thawed and surface sterilized for at least 30 sec in 70% ethanol. When possible, the stomachs were dissected from specimens longer than 1 cm to reduce excess tissues in the sample that may interfere in the analysis. All specimens (or their stomachs) were macerated in a 1.5 mL centrifuge tube filled with 300 μL of 1 × PBS using a sterile, plastic pestle. The specimens were vortexed, solids were centrifuged out over 3 min at 16,100 rcf, and samples were subjected to double-antibody sandwich ELISA. Every other row of 96-well plates (Product number 12-565-501, Fisher Scientific, Denver, CO) were coated with 100 μL of primary antibody (1:500 anti-Rabbit IgG from goat; R-2004, Sigma-Aldrich) in PBS, and incubated for 24 h at 4°C (the other rows were coated with 100 μL of PBS). All subsequent steps were incubated at 23°C. Primary antibody was ejected, each well of the plate was blocked with 360 μL of 1% milk (w/v), and incubated for 30 min. The milk was ejected, and the plate was washed three times with PBS-Tween (PBST; Tween® 20, Product number P1379, Sigma-Aldrich). Each sample (100 μL) was loaded into two wells on a plate, one coated with primary antibody and one coated with PBS. The samples were then incubated for 1 h. Samples were ejected, and the plate was washed three times with PBST. Secondary antibody (anti-rabbit IgG from goat conjugated with horseradish peroxidase; 100 μL in each well; diluted to 1:1000 in 1% milk; Product A-6154, Sigma-Aldrich) was added to each well, and the plate was incubated for 1 h. The secondary antibody was ejected, and the plate washed three times with PBST. TMB solution was added to each well (100 μL), and after incubating for 15 min, the optical density (OD) was read at 630 nm (μQuant, Bio-Tek Instruments, Winooski, VT). On each plate, two 8-well control series accompanied the samples, one with PBS in place of the samples (negative control), and one with Rabbit IgG diluted to 1:500 in place of the samples (positive control). A sample was considered positive if the OD generated in the well that received primary antibody and a given sample exceeded the OD generated in the well that received the sample and PBS (in lieu of the primary antibody) by 2.5 times the standard deviation of the 8-well negative control series (Sutula et al. 1986). Czech Republic samples were analyzed in South Dakota.

### Seed preference assay

Preferences for marked and unmarked seeds were assayed using seeds marked with the two different marking strategies (see above). In the Czech Republic, *Amara aenea*, *A. similata*, *Harpalus affinis*, *H. luteicornis*, *H. rufipes* (Coleoptera: Carabidae) were all offered marked-lyophilized seeds. In the first set of experiments, 20 individual *A. aenea*, *A. similata,* and *H. luteicornis* were offered 15 untreated (control) and 15 treated (marked lyophilized) seeds mounted on plasticine trays (tin trays filled with white plasticine JOVI® (JOVI, Barcelona, Spain), in which seeds were half stuck so that they can be easily acquired by the arthropods; Honěk et al. 2003). *Harpalus affinis* and *H. rufipes* were offered 30 seeds of each type due to larger body size. Trays with seeds were inspected daily and replaced if more than 50% of seeds were eaten. In a separate assay, we examined the preference of *A. aenea* for untreated versus lyophilized but unmarked seeds, using the methodology described above. In South Dakota, we used similar methods to examine the preference of 50 *Gryllus pennsylvanicus* (Orthoptera: Gryllidae) for seeds marked with the filtration method versus unmarked seeds. Each cricket received 75 of each type of seed, which were affixed to the base of the Petri dish arenas using double-sided tape (fine sand was placed over the exposed taped area). In all assays, seed consumption was recorded after 2 days.

### Data analysis

Partial least squares regression analyses were used to determine which taxa (percent positive) were significantly correlated with seed removal for each plot and on each sample period. Separate analyses were used for the recipient (South Dakota) and native (Czech Republic) biota. Taxa included in the model were those that represented >1% of the specimens collected and had at least one individual that tested positive for the seed marker. The residuals were examined and revealed normal distributions. To offer further support for the results of the multiple regression analysis, simple Pearson correlation tests between percent positive of each taxon and seed removal were conducted using the Bartlett chi chi-squared test statistic.

A rarefaction analysis was conducted to estimate the granivore accumulation curves in the native and the recipient biota of dandelion. The abundances of positive specimens in each granivore species were used to calculate this curve (Analytic Rarefaction 1.3). A Chao 1 estimator was calculated for each plot, and resulting mean diversities (S) per plot were used as a further measure of species diversity in the two study locations (Chao 1984). Species representing less than 1% of specimens collected were grouped together at Family-level categories in these diversity analyses. All statistics were conducted using Systat 13 (SYSTAT Software Inc., Richmond, CA).

## Results

### Marker retention

**Figure 1:**
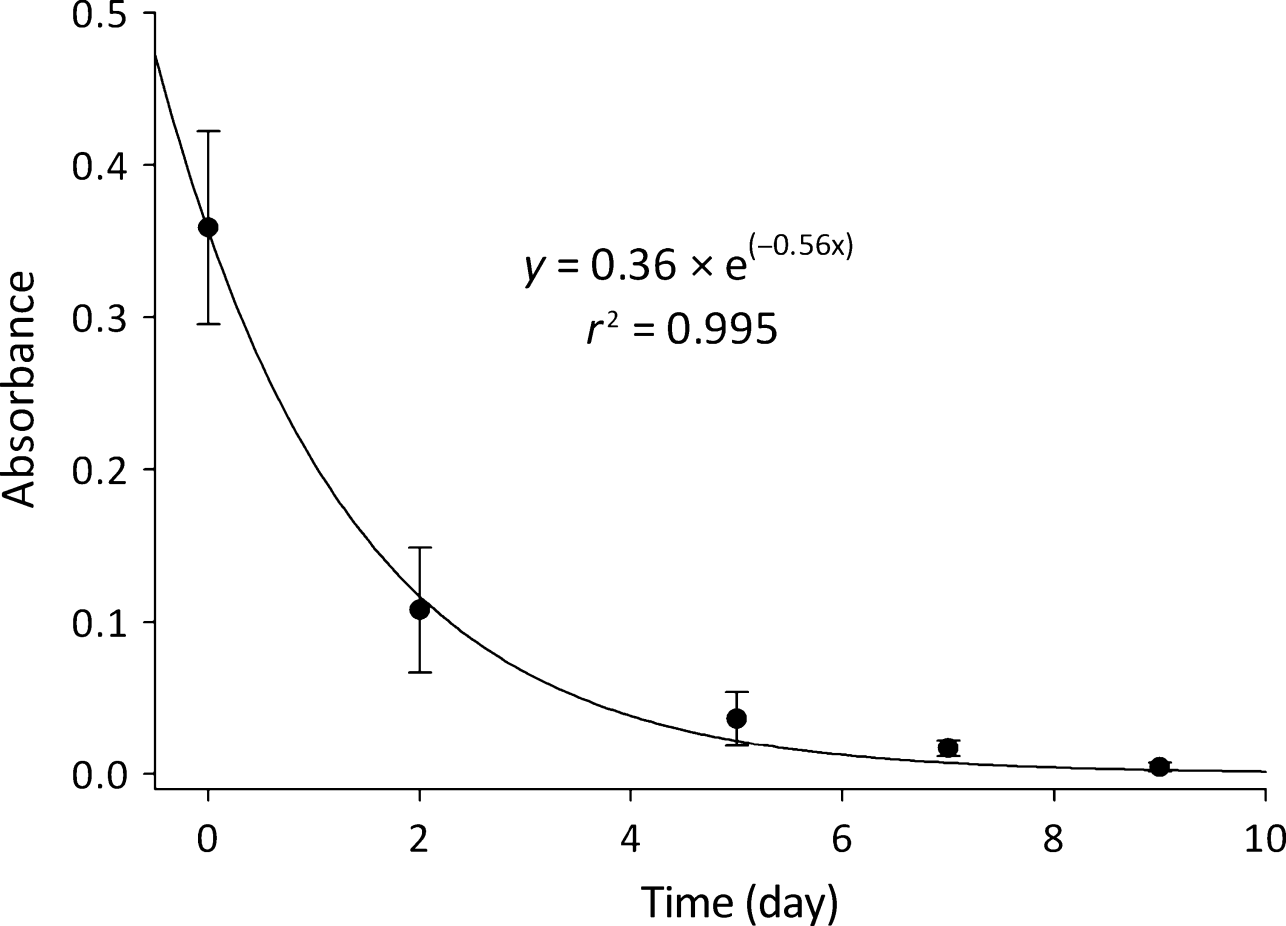
The duration that dandelion seeds remain marked with Rabbit IgG under field conditions. Data points represent mean (SEM) absorbance of the marker remaining on the seeds (*n* = 5 plots per time point), and the relationship is described using a 2-parameter exponential decay curve.

In the field in South Dakota, marked seeds retained a detectable quantity of the marker for at least 10 days, although much of the marker had deteriorated by this time (Fig. 1). Fifty percent of the seed marker was gone within 1.2 days of putting the seeds in the field, and 90% of the marker had been removed or deteriorated within 4.1 days.

### Preference assay for marked and unmarked seeds

The process used to mark the seeds had important implications for whether marked seeds were preferred over unmarked seeds. When the filtration method was used to mark seeds, both the marked and unmarked seeds were consumed equally by crickets (*G. pennsylvanicus*, standardized by insect size) after 48 h in the laboratory (paired *t*-test, *t*_49_ = 0.94, *P* = 0.34). Mean (SEM) consumption rates were 41.36 ± 3.68 and 40.30 ± 3.69 seeds per 48 h for marked and unmarked seeds, respectively. This was the method used in South Dakota. The same pattern was not found for the lyophilized seeds used in the Czech Republic. Three of the five ground beetle species strongly preferred the lyophilized seeds (that were imbibed for 24 h) over the untreated seeds after 48 h. *Amara similata* (*t*_19_ = −5.32, *P* < 0.001; a mean [SEM] of 8.30 ± 1.99 and 17.75 ± 1.74 unmarked and marked seeds consumed, respectively), *Harpalus affinis* (*t*_19_ = −5.81, *P* < 0.001; 10.45 ± 1.50 and 28.45 ± 2.35 seeds) and *Pseudoophonus rufipes* (*t*_19_ = −5.22, *P* < 0.001; 22.65 ± 3.88 and 40.20 ± 3.59 seeds) all consumed significantly more of the marked seeds over the 48 h exposure. Exceptions were *Amara aenea* (*t*_19_ = −1.98, *P* = 0.06; a mean [SEM] 5.80 ± 1.20 and 9.45 ± 1.31 unmarked and marked seeds consumed, respectively), and *Harpalus luteicornis* (*t*_19_ = −1.39, *P* = 0.19; 4.50 ± 1.01 and 6.60 ± 0.93 seeds), which consumed similar numbers of seeds over 48 h. In an extended observation, both of these latter species significantly preferred marked seeds when the total number of seeds consumed over 3 days was compared (*t*_19_ = −2.64, *P* = 0.02). When *A. aenea* was offered unmarked seeds either lyophilized (and imbibed for 24 h) or not, they did not have a preference over 2 days(*t*_19_ = −1.17, *P* = 0.26), but preferred the lyophilized seeds after 3 days(*t*_19_ = −2.55, *P* = 0.02); these results were almost identical to those observed for the protein-marked, lyophilized and un-lyophilized, unmarked seeds. Of these Czech Republic ground beetles used in the preference study, only *A. aenea* was found to have consumed dandelion seeds in the field plots (see below).

### Community description

In South Dakota, a total of 1783 specimens (821 in 2009 and 962 in 2010), representing 65 taxa, were analyzed for the presence of the protein marker. Eighteen species were collected, which each comprised more than 1% of the total number of specimens collected; the rest of species were grouped into family-level categories (Table 1). Even under these conditions, some families represented less than 1% of the taxa and are omitted from Table 1. These were other grasshoppers (Acrididae) (10 individuals from five plots), other crickets (Gryllidae) (17 individuals from seven plots), other camel crickets (Gryllacrididae) (three individuals from two plots), *Orius insidiosus* Say (Hemiptera: Anthocoridae) (one specimen collected), *Otiorhynchus ovatus* L. (Coleoptera: Curculionidae) (five individuals collected in three plots), other beetles (including Scarabaeidae, Lampyridae, and unknown taxa) (13 specimens collected in eight plots), other caterpillars (Lepidoptera) (17 individuals collected in nine plots), other ants (Formicoidea) (15 individuals collected in six plots).

**Table 1:** Community and frequency of dandelion seed consumption in a recipient biota (South Dakota)

Species	Total collected (# plots collected)	Frequency of dandelion consumption (mean ± SEM%)	Coefficient ± SEM, *F*-statistic, *P*-value
Crustacea			
Isopod	35 (8)	39.54 ± 12.88	0.009 ± 0.011; 0.88; 0.35
Diplopoda			
Millipede	45 (9)	50.00 ± 11.38	−0.004 ± 0.011; 0.13; 0.72
Insecta Orthoptera			
*Gryllus pennsylvanicus* Burmeister	55 (7)	37.07 ± 11.93	0.003 ± 0.007, 0.16, 0.70
Gryllidae <0.5 cm	26 (3)	46.83 ± 7.05	−0.007 ± 0.003, 2.31, 0.14
Coleoptera Carabidae			
*Amara littoralis* Mannerheim	33 (10)	33.33 ± 12.91	0.008 ± 0.008, 0.98, 0.33
*Anisodactylus rusticus* (Say)	201 (10)	34.08 ± 4.62	−***0.037 ± 0.008, 18.70, <0.001***
*Bembidion quadrimaculatum oppositum* Say	24 (8)	8.04 ± 6.25	0.008 ± 0.010, 2.40, 0.13
*Cyclotrachelus alternans* (Casey)	146 (10)	5.83 ± 3.25	−0.002 ± 0.004, 0.32, 0.57
*Elaphropus* sp.	24 (9)	4.44 ± 4.44	−0.002 ± 0.002, 0.59, 0.45
*Harpalus compar* LeConte	18 (4)	6.81 ± 6.81	***0.014 ± 0.009, 7.71, 0.008***
*Harpalus herbivagus* Say	22 (7)	26.19 ± 10.82	0.005 ± 0.007, 0.58, 0.45
*Poecilus lucublandus* (Say)	23 (8)	6.67 ± 4.54	0.004 ± 0.004, 3.30, 0.08
*Pterostichus permundus* (Say)	60 (9)	22.45 ± 7.56	0.003 ± 0.004, 0.21, 0.65
Other Carabidae	62 (10)	26.72 ± 6.71	−0.001 ± 0.013, 0.01, 0.93
Curculionidae			
*Spenophorus venatus* (Say)	29 (8)	27.50 ± 12.06	−0.013 ± 0.006, 2.66, 0.11
Staphylinidae			
Other Staphylinidae	23 (6)	3.33 ± 3.33	***0.005 ± 0.005, 8.83, 0.005***
Lepidoptera			
*Malocosoma americanum* (Fabricius)	117 (9)	18.48 ± 10.75	−0.006 ± 0.006, 1.50, 0.23
Hymenoptera Formicoidea			
*Solenopsis molesta* Buren	36 (3)	0	
*Lasius neoniger* Emery	663 (10)	11.58 ± 3.35	−0.011 ± 0.006, 3.42, 0.07
*Formica subsericea* Say	59 (5)	13.67 ± 6.82	0.004 ± 0.005, 0.44, 0.51

Taxa presented represent greater than 1% of the specimens collected in pitfall traps (sample sizes shown). The mean percent of each taxon positive for the seed marker (Rabbit IgG) is calculated per plot. The five most frequent dandelion consumers are indicated with gray shading. The final column presents statistics (regression coefficient for seeds removed ± SEM; the *F*-Statistic, and the *P*-value) from the partial least squares regression analysis (df = 1, 48); significant interactions are indicated with bold italics.

In the Czech Republic, 288 specimens from 20 taxa were analyzed for the presence of the marker. Thirteen of these taxa each represented more than 1% of the total specimens collected (Table 2). Species in the Other Carabidae included *Amara aenea*, *Harpalus luteicornis*, *Pseudoophonus rufipes*, *Pterostichus melas*. Other taxa omitted from Table 2 included Staphylinidae >1 cm in length (two collected in one plot), and *Trachelipus rathkei* (Isopoda) (one collected), and *Lasius niger* (two collected in two plots).

**Table 2:** Community and frequency of dandelion seed consumption in its native range (Czech Republic)

Species	Total collected (# plots collected)	Frequency of dandelion consumption (mean ± SEM%)	Coefficient ± SEM; *F*-value; *P*-value.
Isopoda			
*Armadillidium vulgare* Latreille	64 (3)	74.89 ± 16.17	0.020 ± 0.568; 0.01, 0.94
*Philoscia muscorum* (Scopoli)	10 (1)	20.00	***0.433 ± 0.339, 10.28, 0.007***
Diplopoda			
Julididae	26 (3)	11.43 ± 5.95	−0.106 ± 0.154, 1.76, 0.21
*Polydesmus* sp.	7 (1)	0	
Coleoptera Carabidae			
*Amara convexior* Stephens	13 (3)	7.41 ± 7.41	−0.018 ± 0.110, 0.01, 0.91
*Brachinus explodens* Duftschmid	6 (2)	0	
*Poecilus versicolor* (Sturm)	6 (1)	16.67	0.111 ± 0.330, 1.16, 0.30
Other Carabidae	8 (3)	16.67 ± 23.57	−0.115 ± 0.176, 0.63, 0.44
Curculionidae			
*Barypeithes pullucidus* (Boheman)	25 (3)	10.53 ± 10.53	***0.217 ± 0.163, 8.62, 0.01***
Elateridae			
Elateridae	14 (3)	43.33 ± 23.33	0.037 ± 0.496, 0.02, 0.89
Scarabaeidae			
*Onthophagus joanneae/ovatus* grp.	5 (2)	16.67 ± 25.00	−0.057 ± 0.088, 0.63, 0.44
Staphylinidae			
Staphylinidae	31 (3)	8.33 ± 8.33	−0.027 ± 0.030, 0.13, 0.72
Hymenoptera: Formicoidea			
*Myrmica slovaca* Sadil	65 (3)	9.63 ± 8.26	−0.011 ± 0.045, 0.04, 0.85

Taxa presented represent greater than 1% of the community collected in pitfall traps (sample sizes shown). The mean percent of each taxon positive for the seed marker (Rabbit IgG) is calculated per plot (*n* = 3). The five most frequent dandelion consumers are indicated with gray shading. The final column presents statistics (regression coefficient for seeds removed ± SEM; the *F*-Statistic, and the *P*-value) from the partial least squares regression analysis (df = 1, 13); significant interactions are indicated with bold italics.

### Seed consumption in the field

Dandelion seeds were removed at a rate of approximately 2–4% per day in both South Dakota and the Czech Republic. In South Dakota, seeds were removed from the seed dishes at an overall rate of 0.94 ± 0.11 seeds per dish (3.76% of the seeds; mean ± SEM) on each day of the 7-day observation period. Seeds were removed at a rate of 0.57 ± 0.15 seeds (2.28% of the seeds) over the 1 day exposure period in the Czech Republic. In South Dakota, these rates varied substantially among the observation periods (Dates in 2009: *F*_4,20_ = 17.64, *P* < 0.001. Dates in 2010: *F*_4,20_ = 8.71, *P* < 0.001). In 2009, seed removal rates were 6.76 ± 0.67, 5.04 ± 0.55, 0.32 ± 0.19, 2.32 ± 0.67, and 5.36 ± 0.82 seeds per dish on sample periods beginning on May 22, June 2, 10, 16, and 23, respectively. In 2010, 1.48 ± 0.12, 13.92 ± 2.07, 13.52 ± 2.92, 7.72 ± 0.99, and 9.44 ± 0.97 seeds per dish were removed on sample periods beginning on May 26, June 2, 8, 15, and 22 respectively. Seed removal rates did not vary significantly across sample dates in the Czech Republic (F_4,10_ = 0.67, *P* = 0.63).

**Figure 2:**
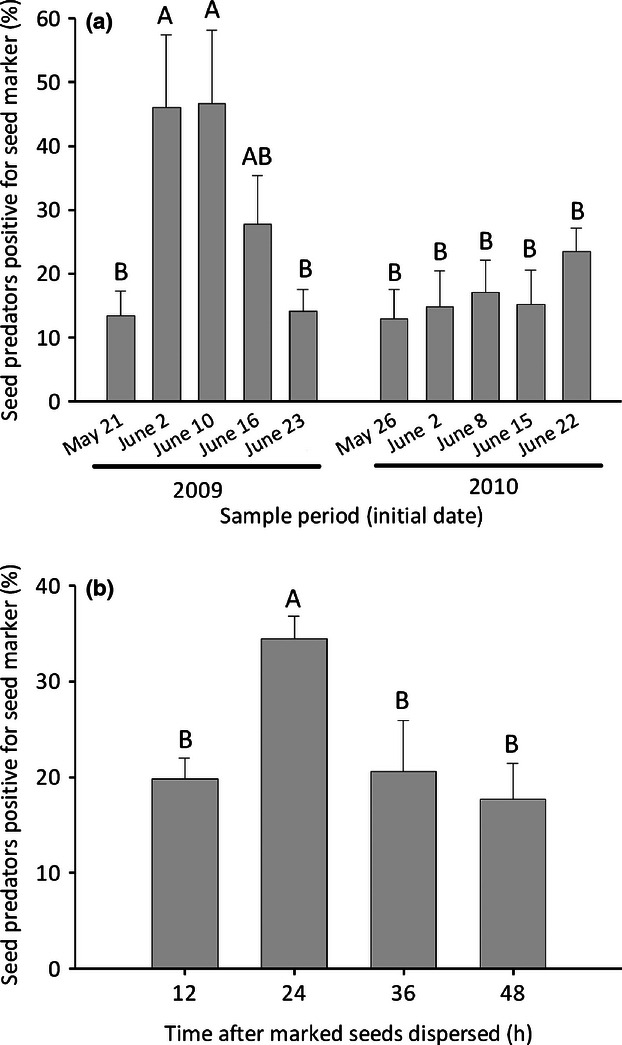
The effects of seasonal sample period (2a) and hours since marked seeds were deployed (2b) on the proportion of the granivore community testing positive for consuming marked dandelion seeds in a recipient biota (South Dakota). Bars topped with different letters are significantly different from one another (LSD means comparison test; α = 0.05).

Gut analysis revealed a high consumption rate of marked dandelion seeds under field conditions. 21.85 ± 3.74% of specimens were positive per plot (pooled across sample dates) for the seed marker in South Dakota. Both sample date and the duration between putting the seeds in the field and when the granivores were collected had significant effects on the proportion of specimens testing positive (sample period: *F*_9,151_ = 6.38, *P* < 0.001; duration since seed deposition: *F*_3,151_ = 5.99, *P* = 0.001; interaction: *F*_27,151_ = 1.34, *P* = 0.14) (Fig. 2). Granivores collected during the first half of June 2009 consumed marked seeds significantly more frequently than over the rest of the observation periods (Fig. 2a). Samples captured 24 h after the marked seeds were available had a significantly higher detection frequency than specimens collected 12, 36, and 48 h after the marked seeds became available (Fig. 2b). In the Czech Republic, 21.60 ± 5.42% of specimens were positive per plot, which was nearly identical to that for South Dakota. However, there were no significant effects of sample period or the duration since seed deposition on the proportion of granivores testing positive for the seed marker in the Czech Republic (sample period: *F*_4,19_ = 0.96, *P* = 0.45; duration since seed deposition: *F*_1,19_ = 0.65, *P* = 0.43; interaction: *F*_4,19_ = 0.62, *P* = 0.65).

**Figure 3:**
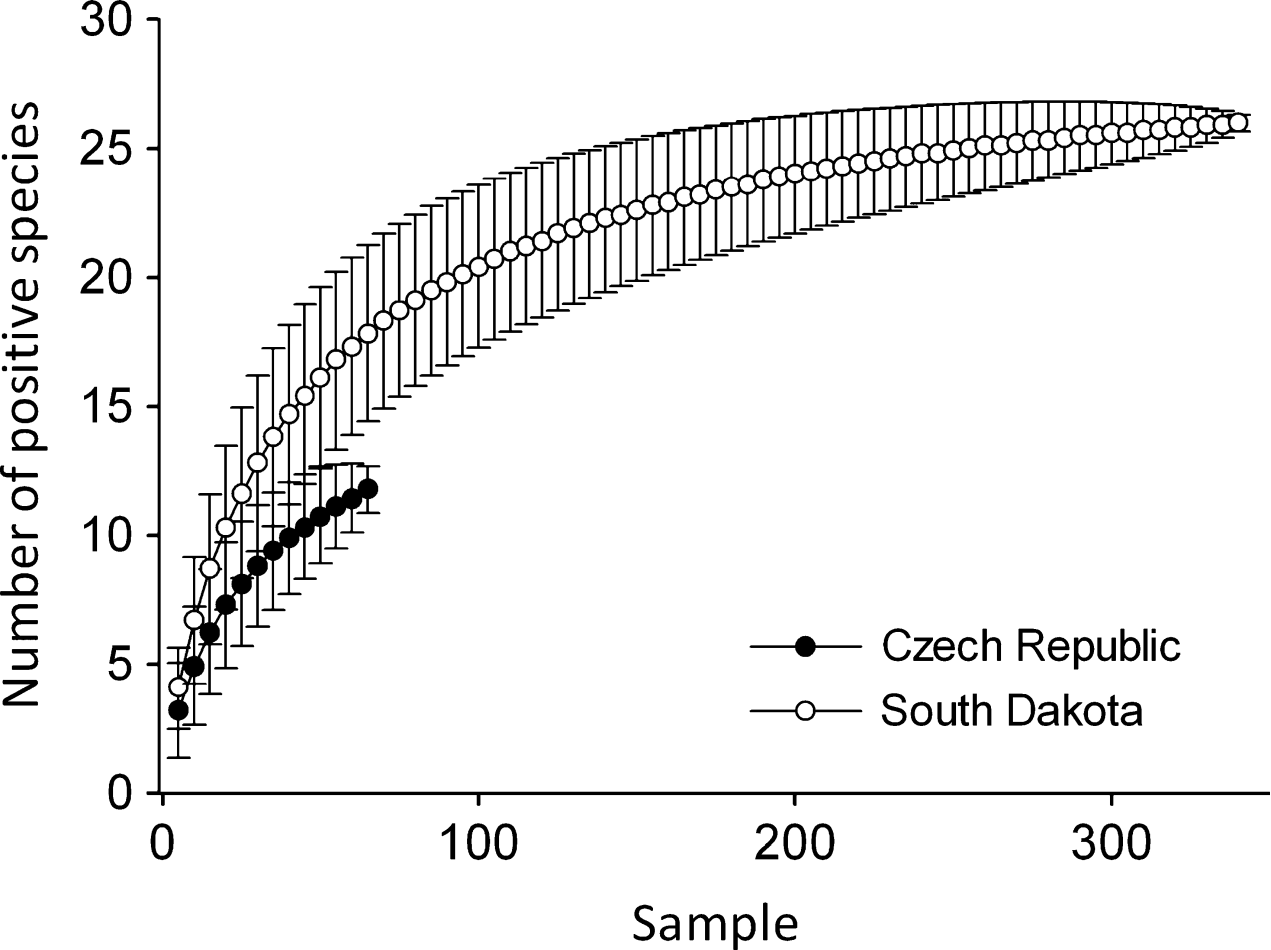
Rarefaction analysis of the granivore communities of dandelion seeds in its native (Czech Republic) and a recipient biota (South Dakota). The number of each species testing positive for the protein marked dandelion seeds was used as the abundance metric in the analysis. Error bars represent the 95% confidence intervals.

A diverse granivore community consumed dandelion seeds in both habitats sampled. Rarefaction analysis revealed nearly twice as many granivores consuming seeds in South Dakota than was found in the Czech Republic (Fig. 3). The Chao 1 estimators confirmed the species accumulation patterns revealed by the rarefaction plots; mean Chao 1 (±SD) estimators per plot for the Czech Republic and South Dakota were 16 ± 18.13 and 27.5 ± 3.76 species.

### Key granivores in the native and recipient biotas

In South Dakota, the partial least squares regression analysis revealed a strong relationship between certain members of the community and seed removal ratios per plot (Table 1). Cumulative variation explained for predictors was 100%, and cumulative variation explained for the responses was 5.11%. Number positive of *Anisodactylus rusticus* was significantly negatively correlated with seed removal rate and numbers of positive Staphylinidae and *Harpalus compar* were significantly positively correlated with seed removal (Table 1). Simple Pearson correlation analysis confirmed the relationships between Staphylinidae and *H. compar* with seed removal rates but the relationship with *A. rusticus* was not corroborated. The Pearson correlation analysis added a significant correlation between numbers positive of *Lasius neoniger* and seed removal rates (Pearson correlation coefficient = −0.31; Bartlett *χ*^2^ = 4.76, *P* = 0.03; a negative correlation) with seed removal that was not revealed by the partial least squares regression. For infrequently collected families not included in Table 1, the mean (SEM) percent positives for the seed marker per plot were: other grasshoppers (20.00 ± 12.25% positive), other crickets (30.16 ± 18.10), other camel crickets (50.00 ± 50.00), *Orius insidiosus* (no positive), *Otiorhynchus ovatus* (33.33 ± 33.33), other beetles (14.58 ± 9.68), other caterpillars (21.11 ± 11.84), and other ants (15.56 ± 10.42).

In the Czech Republic, the partial least squares regression analysis revealed significant relationships between the numbers of some taxa positive and seed removal rates per plot (Table 2). Cumulative variation explained for predictors was 100%, and cumulative variation explained for the responses was 10.41%. The numbers of *Philoscia muscorum* and *Barypeithes pullucidus* positive for the seed marker were significantly associated with seed removal (Table 2). Pearson correlation analysis did not confirm the significant relationship of *B. pullucidus* with seed removal, but did support the interaction of the number of *P. muscorum* positive for the seed marker and dandelion seed removal. Of the other Carabidae collected in the Czech Republic that are not included in Table 2, only one *A. aenea* tested positive for the seed marker. There was one *T. rathkei* that tested positive for the seed marker, and none of the *L. niger* tested positive for the marker.

## Discussion

This study reveals a much more diverse community of granivores than was previously realized for seeds, provides new tools for studying applied and basic questions regarding post-dispersal granivory, and suggests that granivores are fully adapted to dandelion seed resources in the recipient range. Pairing gut content analysis of arthropods with protein-marking of seeds revealed a much more diverse community of post-dispersal granivores than has previously been predicted for a single seed species. Linkage strength of particular granivores did not always correlate well with seed removal rates, potentially due to the fact that relative densities of these taxa were not well measured by pitfall traps. The method of seed marking thus has merit as a tool used in conjunction with other methods (e.g., seed removal, laboratory feeding trials, etc.) to help resolve which species are potentially important granivores of plants.

Many members of the diverse communities of post-dispersal granivores are polyphagous, consuming other foods in addition to a given seed species. The percentage of the overall communities that tested positive for the marker was very similar in the two regions (around 22% of granivores tested positive), but apparently this granivory was inflicted by a more diverse community in the recipient biota (although sampling intensities varied between the sites). This is in spite of lyophilizing the seeds in the Czech Republic range, which may have increased their attraction to granivores. Many of the taxa that consumed marked dandelion seeds are polyphagous omnivores, some of which are likely food limited, and may switch or expand their diets in response to local resource availability like local seed rain (Frank et al. 2011; Lundgren and Harwood 2012). Granivory of a particular seed species depends on relative resource availability in the habitat, attributes of the granivores, and the morphological, physiological, and defensive properties of the seed themselves (Lundgren 2009). Clearly in the case of this study system, dandelion seeds were palatable to a wide variety of arthropods.

This study reveals a more taxonomically diverse post-dispersal granivore community than has ever been described before. The use of gut analysis to establish trophic linkages to a particular seed substantiates that commonly identified granivores such as ants, carabids, and crickets are important granivores, but expands the list to include certain species of caterpillars, weevils, and rove beetles, which have largely been ignored previously. The study also suggests that isopods and millipedes are important seed consumers in both the recipient and native biota (as was seen by Saska 2008a; Koprdová et al. 2010). This study suggests that some “carnivorous” species (e.g., *Bembidion quadrimaculatum*, *Cyclotrachelus alternans, Elaphropus* sp., *Poecilus lucublandus*, *Poecilus versicolor*, and *Pterostichus permundus*) are more polyphagous than was believed (our experience with gut analysis leads us to believe that it is unlikely that these predators ate a granivore that just consumed a marked seed), and has important implications on where these species fit within food webs.

Gut content analysis of pitfall-collected arthropods did not consistently correlate with seed removal rates. Of the top five strongest trophic linkages between dandelion seeds and granivores in the recipient and native granivore communities, only the isopod *Philoscia muscorum* was positively correlated with seed removal rates (in the Czech Republic). One potential explanation for the disconnect between strong trophic linkages revealed by gut analysis and the impact that these taxa had on seed removal rates was that our sampling scheme (pitfall trapping) did not reflect the actual densities of these taxa (Topping and Sunderland 1992; Lang 2000). An abundant arthropod taxon with a weak trophic link to dandelion seed could conceivably have a greater impact on seed removal than a rare taxon with a strong trophic link. Some have demonstrated strong correlations of seed removal with particular granivore taxa using pitfall trapping (Davis and Liebman 2003), while others have not (Saska et al. 2008). Regardless, obtaining actual densities of the now recognized diverse granivore community would add to our understanding on the relative contributions of these taxa to seed removal.

The use of protein marking of seeds offers opportunities for understanding the structure of seed-associated food webs, but there are constraints to the method that also need to be recognized. First, this work clearly demonstrates that the method for applying the marker affects the preferences of the granivore (although it is important to note that different taxa were used to test the two marking methods in this study). Care must be taken not to weaken the seed coat or imbibe the seed, as the strength of the testa constrains which arthropods will successfully consume the seed (Cardina et al. 1996; Lundgren and Rosentrater 2007). The duration of marker stability in the environment and in the insect gut are important considerations for designing experiments using this technique. Ninety percent of the marker is removed from the seed within 4 days of environmental exposure, so targeting arthropod collections within the first few days following seed deployment is advisable. Method development work suggests that the marker was detectable in the stomachs of the carabid beetle *Harpalus pensylvanicus* De Geer for up to 8 h after feeding (J. G. Lundgren unpubl. data), which is consistent with other work on marker stability within insect stomachs (Hagler 2011). The trophic linkages revealed by this method should be conservatively interpreted because the method gives only a sample of the taxa that have eaten the marked seed within a few hours of being collected and frozen. In the end, the paired use of gut analysis and protein marking of seeds is a powerful tool that is best used in concert with other methods (seed removal, population monitoring, etc.) to get a more complete picture of the dynamic interactions of seeds and their granivore community.

The work on post-dispersal granivory of an invasive plant suggests that granivore communities are a diverse group including what are traditionally regarded as seed specialists as well as polyphagous arthropods. These soil arthropod communities are shaped by biotic and abiotic characteristics of a habitat, and can be managed to inflict greater effects on a target pest. As such, conserving a healthy and diverse soil arthropod community that can adapt to and resist seedling establishment of exotic plants may be an important component of greater management strategies for curtailing the harm caused by these invasives.
